# Growth rate evolution in improved environments under Prodigal Son dynamics

**DOI:** 10.1111/eva.12403

**Published:** 2016-09-28

**Authors:** Sinéad Collins

**Affiliations:** ^1^ Institute of Evolutionary BiologyUniversity of EdinburghEdinburghUK

**Keywords:** cell division rate, *Chlamydomonas*, CO_2_, environmental change, individual‐based model, microevolution, *Ostreococcus*, oxidative damage, primary production

## Abstract

I use an individual‐based model to investigate the evolution of cell division rates in asexual populations under chronic environmental enrichment. I show that maintaining increased growth rates over hundreds of generations following environmental improvement can be limited by increases in cellular damage associated with more rapid reproduction. In the absence of further evolution to either increase damage tolerance or decrease the cost of repair or rate of damage, environmental improvement does not reliably lead to long‐term increases in reproductive rate in microbes. Here, more rapid cell division rates also increases damage, leading to selection for damage avoidance or repair, and a subsequent decrease in population growth, which I call Prodigal Son dynamics, because the consequences of ‘living fast’ force a return to ancestral growth rates. Understanding the conditions under which environmental enrichment is expected to sustainably increase cell division rates is important in applications that require rapid cell division (e.g. biofuel reactors) or seek to avoid the emergence of rapid cell division rates (controlling biofouling).

## Introduction

The trade‐off between producing high‐quality offspring and many offspring is well studied and provides a general explanation for why the optimal rate of offspring production is often below the maximum possible rate (Flatt and Heyland 2011). However, with microbes, studies of the quality vs. quantity of offspring trade‐off are limited to looking at major shifts in life‐history strategies, such as the evolution of asymmetric cell division (Chao 2010; Clegg et al. 2014; Franklin 2014), or at the macroevolutionary transition to specialized germ and soma (Goldsby et al. 2014). I suggest that this trade‐off is at the crux of understanding growth rate evolution within cell division and life‐history strategies for unicellular microbes and that limits on the ability to produce high‐quality offspring is a key driver of microbial growth rate evolution in rich environments. While the recent work above has considered that the ability to dilute or repair cellular damage can drive the evolution of asymmetric cell division and repair in unicells, the microevolutionary consequences of damage accumulation in unicells have yet to be investigated in terms of growth rate evolution.

Microbial evolution experiments and theory focus on environmental deterioration, and largely neglect environmental improvement, where the absolute rate of cell division increases following an environmental change. In most studies, evolution in an initially suboptimal environment usually involves the full or partial recovery of absolute ancestral growth rates before environmental deterioration (Elena and Lenski 2003; Buckling et al. 2009). However, environmental enrichment is one of the hallmarks of global change and urbanization in aquatic systems (Howes et al. 2015), so that evolution in environments where nutrient limitation is relaxed has the potential to occur in natural populations, and has the potential to drive changes in life‐history trade‐offs (Snell Rood et al. 2015). For example, CO_2_ enrichment is one common component of global change (Gruber 2011). Photosynthetic unicells such as *Chlamydomonas reinhardtii* photosynthesize more quickly and produce more peroxide under high CO_2_ conditions, but do not increase peroxide breakdown proportionally (Mettler et al. 2014; Roach et al. 2015). Importantly, CO_2_ enrichment often occurs in the absence of environmental deterioration, and, in cases where fertilizer or other nutrient‐rich runoff is also present in aquatic systems, CO_2_ enrichment can occur alongside other types of nutrient enrichment (Boyd and Hutchins 2012; Snell Rood et al. 2015). Because of this, understanding how microbes will evolve under global change requires expanding our studies of growth rate evolution to include environmental improvement as well as environmental deterioration. In addition, improving our ability to use microbes for production purposes, for example, in biofuels, requires understanding the conditions under which rapid cell division rates and high‐biomass populations can be indefinitely sustained (Raven and Ralph 2014; Sarkar and Shimizu 2015). Controlling, or at least predicting, microbial growth in rich environments (e.g. reducing or predicting biofouling) may be helped by an improved understanding of what conditions might preclude sustained rapid growth of microbial populations.

**Figure 1:**
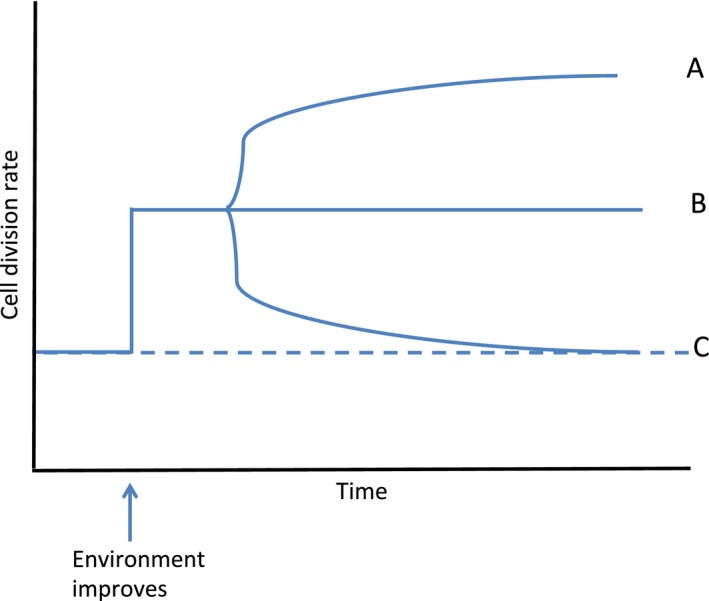
Cartoon showing three possible responses to environmental improvement. Following environmental improvement, growth rate initially increases as the result of a plastic response, after which three possible scenarios can occur over hundreds of generations: (A) adaptive evolution may occur, allowing even higher growth rates than possible with the plastic response alone (no Prodigal Son dynamics); (B) the growth rate associated with the adaptive plastic response may be sustained, perhaps with some genetic accommodation, or (C) the plastic increase in growth may be reversed, showing Prodigal Son dynamics.

My motivation for exploring growth rate evolution in improved environments is based on a pattern that has emerged from several microbial evolution experiments studying phenotypic evolution in photosynthetic microbes in high CO_2_, mainly in the laboratory or in enclosures (see Fig. 1). Studies with *C. reinhardtii* have shown that after hundreds of generations of evolution in high CO_2_, evolved growth rates never exceed the ancestral plastic response (Fig. 1, scenario B), but that the ability to induce high‐affinity carbon uptake disappears (Collins et al. 2006). This is consistent with growth patterns of microalgae isolated from high CO_2_ springs and other noncalcifying species evolved at high CO_2_ (Collins and Bell 2006; Low‐Décarie et al. 2013). This loss of high‐affinity carbon uptake is remarkable since *C. reinhardtii* and most other photosynthetic microbes have an inducible carbon concentrating mechanism (CCM) that takes up inorganic carbon and concentrates it near the carbon fixing machinery (Raven et al. 2012). *Ostreococcus* sp., on the other hand, have a plastic response to CO_2_ enrichment where cell division rates increase, but lineages often reverse this response after hundreds of generations in a high CO_2_ environment (Schaum and Collins 2014; Fig. 1, scenario C). *Ostreococcus* sp. cells that fail to lower their growth rates in high CO_2_ environments after hundreds of generations have lower mitochondrial potential and are less able to withstand heat shock than lineages that have lowered their growth rates in high CO_2_ (Schaum et al. 2015). High‐CO_2_‐evolved *C. reinhardtii* cells have mitochondria that may differ in size and potential relatively to ambient CO_2_‐evolved cells (S. Collins and M. Brickley, unpublished data). Both *C. reinhardtii* and *Ostreococcus* sp. evolved at high CO_2_ are poor competitors, and competitive ability is negatively correlated with population growth rate (Collins 2011; Schaum and Collins 2014). In addition, growth at ambient CO_2_ is often reduced or arrested in both taxa, such that the tolerance of high‐CO_2_‐evolved lineages to further changes in CO_2_ is reduced relative to their ancestors (Schaum et al. 2015). Few evolution experiments with photosynthetic microbes and environmental improvement exist, but those that do are consistent in that no direct response to selection, measured as an improvement in growth rate in excess of the plastic response (Fig. 1, scenario A) is observed, unless there is an initial decrease in growth (Collins et al. 2013). However, in cases where evolution occurs in multigenotype assemblages, there is evidence of genotype sorting, suggesting that natural selection occurs in response to CO_2_ enrichment (Low‐Décarie et al. 2013; Scheinin et al. 2015). Based on these phenotypic data, we proposed that increases in cellular damage were driving the evolution of slower growth rates in CO_2_‐enriched environments when it was observed and that this damage could explain both the lower ability of cells to tolerate environmental change and the lower competitive ability of high‐CO_2_‐evolved cells (Schaum et al. 2015). Here, I explore a general explanation for how cellular damage and repair affect growth rate evolution in microbes subjected to environmental improvement.

My reasoning is as follows. Many metabolic processes, including photosynthesis, are sensitive to nutrient enrichment, and increasing the rate of metabolism can increase the rate of production of molecules that can damage cells in various ways ranging from oxidizing DNA to producing proteins with reduced function (Fischer 2006; Glaeser et al. 2011). By raising metabolic rates to divide faster, unicells that cannot simultaneously increase repair rates pass on more damaged components to daughter cells, which then have a lower probability of survival and reproduction. Because unicells do not have a dedicated germ line, damage can accrue over generations. While asymmetric cell division can increase the threshold of damage that can be tolerated, it cannot remove the threshold altogether (Franklin 2014). Thus, if we extend the argument over many asexual generations, lineages that grow faster at the cost of accumulating damage are more likely to eventually go extinct than lineages that grow more slowly and avoid accumulating damage, either through devoting more resources to repair mechanisms at the expense of rapid reproduction, or by avoiding damage by keeping metabolic rates low, again at the expense of rapid reproduction. This leads to the intriguing possibility that when increased damage accrues due to increases in the rate of cell division, and adaptive evolution that allows this damage to somehow be controlled or tolerated does not occur, slower‐growing lineages can eventually emerge to replace their faster‐growing ancestors by producing fewer, higher‐quality daughter cells. In this way, populations in enriched or improved environments can evolve substantially, first through rapidly reproducing genotypes replacing their slower‐growing ancestors, followed by the fast growers accruing damage. Then, slower‐growing genotypes can appear by mutation (or immigration) and replace the now damaged fast growers (Fig. 1, scenario C). I hypothesize that this is what occurred during the *Ostreococcus* sp.high CO_2_ evolution experiments. I call this ‘Prodigal Son’ dynamics, where although a great deal of change in phenotype (lifestyle) occurs following enrichment, profligate use of resources to live fast ultimately forces a return to the ancestral phenotype (lifestyle), despite a stable rich external environment. In short, substantial adaptive evolution occurs following an environmental change, but initial changes eventually become detrimental, causing the re‐evolution of many ancestral trait values. In contrast, lineages that evolve ways to repair or avoid damage at higher growth rates would avoid Prodigal Son dynamics, thus maintaining rapid growth, which is what I hypothesize was seen in the *Chlamydomonas* experiments.

## Simulation

I tested the Prodigal Son hypothesis using an individual‐based simulation to see how reductions in viability due to damage incurred by rapid cell division affected the evolution of cell division rates when populations evolved in an enriched environment. As explained in the introduction, this is motivated by data where photosynthetic unicells that respond plastically to CO_2_ enrichment by increasing their cell division rates. The model does not preclude (or require) a plastic response to environmental improvement, but is only concerned with a response that is sustained over hundreds of generations. No particular genetic or epigenetic mechanism is assumed.

I explore the conditions under which Prodigal Son dynamics can or cannot occur by simulating a population responding to environmental enrichment under different parental contributions to cellular damage levels, tolerances to cellular damage, and costs of repairing damage. By exploring different relationships between traits that I have observed evolving in experimental populations (growth rate and evidence for damage), I show the conditions under which Prodigal Son dynamics can occur or be avoided. Because the model explores the consequences of the relationship between cell division rates and cellular damage following environmental enrichment, it can be used to generate testable hypotheses on changes in measurable traits in future experimental populations.

The simulation uses an asexual population of fixed size. A general walkthrough of the simulation is given first, with a detailed explanation following. Briefly, the model explores the evolution of cell division rates (or number of viable daughter cells produced per parent cell) where some proportion of parental damage is passed on to offspring and when processes generating damage also increase with the rate of cell division. The population evolves under selection for higher absolute growth, in an environment that is enriched (where the maximum growth rate allowed is higher). However, increasing growth rate increases the damage incurred by individuals, which is in turn passed on to their offspring, which increases the chance that the offspring is nonviable or infertile (the two are equivalent in the model).

A single round of the simulation proceeds as follows. Each generation, the population is sampled randomly weighted by growth rate, such that individuals who divide faster have a higher chance of contributing offspring to the next generation. Sampled individuals have a probability of either producing viable offspring or not, depending on their level of damage, with higher levels of damage leading to lower probabilities of viable offspring. If an individual produces viable offspring, the offspring then undergoes mutation that affects its growth rate. Offspring damage is calculated from a combination of damage inherited from the parent and damage resulting from offspring growth rate. Sampling continues, with replacement, until the entire population is replaced. Thus, faster‐growing individuals have a higher probability of contributing offspring than do slower‐growing individuals, although these offspring may have lower chances of being viable.

The Prodigal Son hypothesis suggests that increasing growth in an enriched environment incurs damage from an increase in metabolism and that some proportion of this damage is inherited by daughter cells. I model a case where both parental and offspring growth rates can contribute to the total damage in offspring. Offspring damage is calculated from inherited parental damage and from damage due to offspring growth. I first explain how damage due to offspring growth is modeled, then show how inherited damage from parental cells is incorporated.

Damage incurred due to growth is a function of the rate of damage of a cell and the rate of repair of a cell, such that

(1) $$\frac{dD}{dt}=gD_{\max}-RD$$

where *D *= damage, *t* = time in generations, *D*_max_ = maximum rate of damage, *g *= growth rate. Growth rates are real numbers. In the ancestral environment, *g* ∈ [0, 1], and in the enriched environment, *g* ∈ [0, 2]. *R* is a function of maximum and minimum repair rates such that

(2) $$R=R_{\max}\left(1-g\right)+R_{\min}g$$

where *R*_max_ is the rate of repair when all resources are devoted to repair and *R*_min_ is the minimum rate of repair possible. A cutoff function ensures that growth rates do not go beyond the defined rates. In both cases, a linear trade‐off between allocating cellular resources to growth and repair is assumed, although the slopes of the linear function need not be the same for repair and damage. This is in line with Clegg et al. (2014), which argues that repair and growth should be linearly related, as resources diverted to building, maintaining, or fueling repair machinery (such as enzymes and ATP) cannot be used for growth. While other shape trade‐offs are certainly possible, few data are available, so I use a linear trade‐off for simplicity. From previous empirical work examining the evolution of slowed growth in high CO_2_ environments in the unicellular alga *Ostrococcus* sp., we see that rhodamine123 fluorescence, which correlates positively with mitochondrial potential, is lower in lineages that have maintained high growth for hundreds of generations in the enriched environment and higher in lineages that have lowered their growth (Schaum and Collins 2014). This is consistent with there being a trade‐off between maintaining mitochondrial function, perhaps through minimizing oxidation, and maintaining rapid cell division rates. Several other traits indicative of generally damaged cells are present in the fast‐dividing populations, including reduced tolerance to heat stress and lower responsiveness to competitors, were reported in the same study.

The equilibrium damage of a cell when damage is due only to growth, *D*_eq_ at a given g is calculated by setting d*D*/d*t* = 0 and is given by

(3) $$D_{\mathrm{eq}}=\frac{\left(D_{\max}g\right)}{R}$$

This assumes that damage increases linearly with growth rate. Most microbial models to date assume that damage accumulates at some constant rate that is not explicitly a function of growth (although the dirty work hypothesis allows for different mutation probabilities, where higher mutation probabilities could be interpreted as higher metabolism associated with faster growth; Chao 2010; Clegg et al. 2014; Franklin 2014; Goldsby et al. 2014). Although numerous theories of aging, including the oxidative theory of aging, posit that increased metabolic activity leads to decreased cellular function because of damage, data on the form of the relationship between cell division rates and damage do not exist.

The projected damage (*D*_Δ*t*_) for a cell incorporates both damage inherited from a parent and damage due to growth. This is calculated by assuming that the initial damage in the daughter cell, immediately after it separates from the parent, is equal to *D*_p_, the level of damage of the parent cell (this can easily be set to 0.5*D*_p_, or indeed any other value, but this does not change the behavior of the model). Thereafter, the metabolic rate of the daughter cell modifies the amount of damage over some time Δ*t*. Different values of Δ*t* correspond to different proportions of parental and offspring control over offspring damage. When Δ*t* = 0, damage is determined entirely by the parent and as Δ*t* goes to infinity, damage is determined entirely by the daughter cell, where for a given *g*

(4) $$D_{\Delta t}=D_{\mathrm{eq}}+\left(D_{p}-D_{\mathrm{eq}}\right)e^{\left(R\Delta t\right)}$$

Now that cellular damage is linked to both parent and offspring growth, it can be used to calculate the probability that a cell is not viable (probability of death = *V*) as a Hill function where

(5) $$V=\frac{D^{\alpha }}{\left(KD^{\alpha }+D^{\alpha }\right)}$$

where *α* is a shape parameter for the Hill function, where high values of *α* correspond to a steeper function, and *K* is the damage level where *V* = 0.5. The value of *K* evolves in the ancestral environment and then remains fixed in the new environment. I explore the consequences of *K* evolving in the Results section.

Note that if there is no parental contribution to damage (very large Δ*t*), then equilibrium growth rate is

(6) $$(1-VD_{\mathrm{eq}})g$$

**Figure 2:**
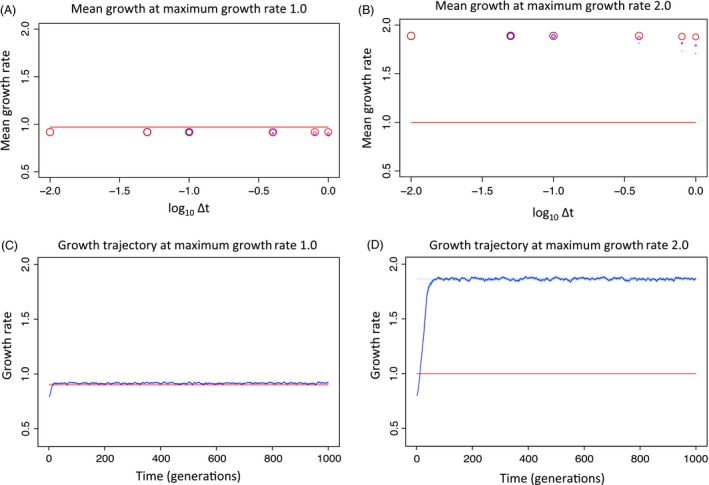
Growth rates over 1000 generations for populations of 1000 individuals that in constant environments with a maximum possible growth rate of 1.0 (A and C) or a maximum possible growth rate of 2.0 (B and D). Panels (A) and (B) show mean growth rates after 1000 generations over values of Δ*t* (values are 0.01, 0.05, 0.1, 0.4, 0.8, 1.0), with lower values of Δ*t* indicating lower contributions of offspring growth to damage and higher values indicating higher contributions of offspring growth to damage. Different steepnesses of the Hill function relating damage to mortality risk are indicated by different colors. Blue: *α* = 2, purple: *α* = 4, red: *α* = 10. Point sizes are inversely proportional to the number of offspring mortality events over the simulation, such that larger points have suffered fewer deaths (have a higher chance of persisting rather than going extinct). For all Figs 2A, B, 3, and 4, each point is the result of 100 independent runs of the simulation at a given set of parameter values. Horizontal red line indicates the predicted fitness at equilibrium in an environment where the maximum growth rate is 1.0 when damage depends entirely on offspring growth rate. Panels (C) and (D) show representative individual simulations of growth rate evolution over 1000 generations. Dark blue line indicates mean growth rate. Light blue shadow indicates variance in growth rate within the population.

**Figure 3:**
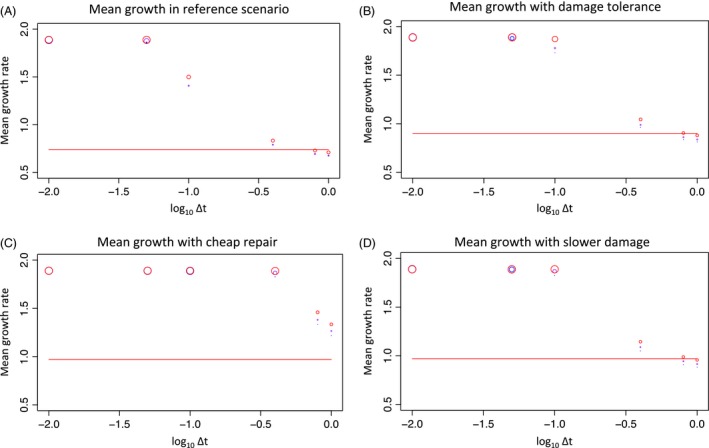
Mean growth rates for populations that evolve for 1000 generations in an enriched environment with a maximum possible growth rate of 2.0. (A) Reference scenario where the damage tolerance, repair and damage rates do not change between standard and enriched environments; (B) cost of repair is lowered in rich environment relative to standard environment; (C) damage tolerance is increased in rich environment relative to standard environment; and (D) the rate of damage increase per unit increase in growth is slowed in rich environment relative to standard environment. The *x*‐axis shows different values of Δ*t*, with lower values indicating lower contributions of offspring growth rate to damage and higher values indicating higher contributions of offspring growth rate to damage. Different steepnesses of the Hill function relating damage to mortality risk are indicated by different colors. Blue: *α* = 2, purple: *α* = 4, red: *α* = 10. Horizontal red line indicates the predicted growth rate at equilibrium when damage depends entirely on offspring growth rate. Point sizes are inversely proportional to the number of offspring mortality events over the simulation, such that larger points have suffered fewer deaths (have a higher chance of persisting rather than going extinct).

**Figure 4:**
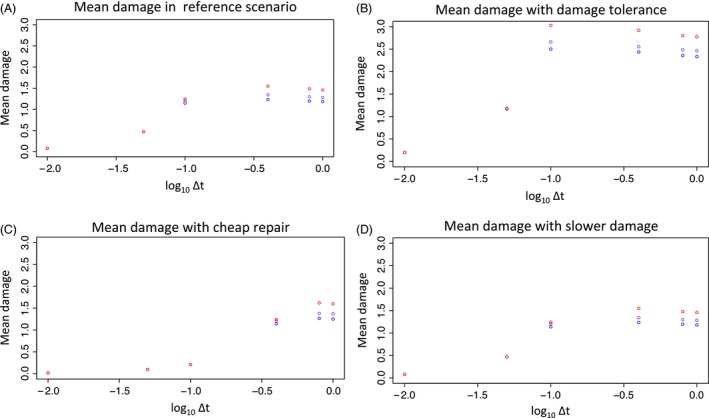
Mean damage levels per cell for populations that evolve for 1000 generations in an enriched environment with a maximum possible growth rate of 2.0. As shown in Fig. 3, (A) Reference scenario where the damage tolerance, repair and damage do not change with environmental enrichment; (B) cost of repair is lowered in rich environment; (C) damage tolerance is increased in rich environment; and (D) the rate of damage increase per unit increase in growth is slowed in rich environment. The *x*‐axis shows different values of Δ*t*, with lower values indicating lower offspring contributions (higher parental contributions) to damage and higher values indicating higher offspring contributions (lower parental contributions) to damage. Different steepnesses of the Hill function relating damage to mortality risk are indicated by different colors. Blue: *α* = 2, purple: *α* = 4, red: *α* = 10.

Simulations were run with a maximum growth rate of 2.0 in the enriched environment, with initial damage loads and equilibria calculated assuming that the ancestral environment allowed a maximum growth rate of 1.0, using a population size of 1000 individuals for 1000 generations. This leads to variances in growth on the order of 10^−5^–10^−4^ and variances in damage on the order of 10^−8^–10^−4^ for the mean values seen in Figs 2, 3, 4. Mean values are calculated in all cases from 100 independent runs of the simulation per set of parameter values. In all cases, I use an ancestral environment where, arbitrarily, the damage rate is set at 5, the maximum repair rate is 5, and the mutation rate is 0.05. Evolved populations are characterized by their growth rate, damage levels, and the number of offspring mortalities that occurred over the course of the simulation (equivalent to the number of selective deaths that occurred over the entire simulation). Simulations were done over several values of *α* (steepness of the Hill function relating damage to the chance of mortality) and Δ*t* (contributions of parental damage versus offspring growth rate to offspring damage). R code is available as Supplementary Material (Data S1).

## Results

I first show that damage can limit growth rate evolution under environmental improvement, demonstrating Prodigal Son dynamics in the absence of evolution in damage and repair strategies over a range of values of Δ*t* (parental versus offspring control over damage) and *α* (steepness of Hill function determining the relationship between damage and mortality). In general, I find that population growth is more limited by damage as Δ*t* increases and offspring growth contributes more to cellular damage and that the number of selective deaths needed for adaptation also increases, suggesting that extinction rather than escaping Prodigal Son dynamics could occur when cellular damage is largely determined by offspring growth rate. I then consider evolutionary strategies for escaping Prodigal Son dynamics by evolutionary changes to damage or repair strategies in the following ways: increasing *K*, the level of damage where the chance of mortality is 0.5 (damage tolerance), reducing the slope of the relationship between repair and growth (cheaper repair), and lowering the amount of damage incurred by increasing growth (damage reduction). The reasoning behind these as well as the equations they alter is given below. All of these strategies allow populations to escape Prodigal Son dynamics for some values of Δ*t* and *α*, but none allow escape over all values of Δ*t* and *α*. In all cases, the efficacy of escape strategies depends critically on Δ*t*.

When the simulation is run for a population in a stable environment and no enrichment occurs (maximum growth rate = 1.0; Fig. 2A), The population grows at near the maximum growth rate over all values of Δ*t*. This is true for a stable environment with a higher maximum growth rate as well (maximum growth rate = 2.0; Fig. 2B). In both cases, the slightly lower growth rate is due to the baseline cost of repair. Populations reach a growth rate of 0.91 ± 0.00017 (average ± variance over all Δ*t* and all *α*) in the first environment and 1.85 ± 0.0033 in the second. Representative single runs of the simulation are shown in Fig. 2C and D. This demonstrates that when the environment is stable and the population evolves damage–repair equilibrium values in that same environment, the population can grow near the maximum possible growth rate.

I model environmental improvement by calculating the equilibrium damage–repair values in the first environment, where the maximum possible growth rate was 1.0, and then raising the maximum possible growth rate to 2.0. The rate of damage accumulation, repair rate, and damage tolerance of the populations do not vary with environment or evolve. I refer to this as the ‘reference scenario.’ The characters of populations evolved in an environment where the maximum growth has been increased from 1.0 to 2.0 are shown in Figs 3A and 4A. The maximum evolved growth rate is 1.89 ± 5.57 × 10^−5^ (growth rate ± variance at Δ*t* = 0.01, *α *= 4), which is set by damage–repair equilibrium, and is only reached in evolved populations when offspring growth rate has little effect on damage because Δ*t* is low (0.01 or 0.05) – here offspring damage is primarily determined by parental damage, so that offspring growth can increase by mutation over time, but damage will not increase much, since ancestral damage continues to be inherited. As Δ*t* increases, the evolved growth rates decrease. When Δ*t* is 0.4 or larger, growth rates are lower in the improved environment than in the ancestral environment because individuals with growth rates near the ancestral equilibrium value have a higher chance of producing faster‐growing (and thus more damaged and less viable) offspring than did their ancestors, as the upper limit on growth has been increased. In this case, growth rate actually decreases when an environmental change allows for more rapid growth when offspring growth rates contribute substantially to cellular damage.

The reference scenario assumes that strategies for dealing with damage and repair cannot evolve. This is unlikely to be the case. Given that damage and repair strategies can evolve, there are then three nonexclusive ways that they may do so that could allow cell division rates to increase: Cells may become more damage tolerant, the cost of repair can decrease, or the damage incurred per increment of growth gained can decrease. I explore these different possibilities below. Because there are no data on the shapes of the relationships between repair and growth or damage and growth, and extremely limited data on the shape of the relationship between damage and mortality (Zakrzewska et al. 2011), I limit my interpretation to qualitative shifts in population‐level characters relative to the reference scenario. Comparing the outcomes of changing damage tolerance, repair, or damage rate directly will depend on the shapes of those relationships with growth. While my choices are consistent with other theoretical work on the evolutionary impacts of cellular damage, speculating on the relative values of the evolved populations by competing them is not useful until there are empirical data on the shapes of the individual relationships. To compare the evolved populations quantitatively in a way that informs our understanding of the biology being modeled, data are needed on the relationships between damage rate, damage tolerance, and repair rates with growth, as well as the evolvability of those relationships.

If the new environment is more permissive, it may be possible for cells to tolerate more damage and still be viable. I explore this by increasing *K*, the level of damage where the chance of mortality is 0.5 (see eqn (5)). Damage tolerance in an enriched environment is shown in Figs 3B and 4B. If *K* is increased *D*_eq_/1.1 from the reference scenario value of *D*_eq_/2, then growth increases for all levels of parental contributions to offspring damage (at all Δ*t*) relative the reference scenario. In the reference scenario, growth is maximum at only the two lowest values of Δ*t* (0.01 and 0.05), whereas in damage tolerant cells, maximum growth also occurs at Δ*t* = 0.1. As expected, the mean damage level per cell increases over all Δ*t* when damage tolerance is increased. The evolution of this strategy could be measured in experimental populations if viable cells evolved in rich environments were, on average, more damaged than viable cells evolved in a reference environment.

If, on the other hand, repair requires a smaller proportion of cellular resources in the improved environment than in the ancestral environment, cells may be able to repair the extra damage associated with more rapid reproduction. This represents a scenario where the new environment allows cells to redirect resources, perhaps by downregulating machinery such as CCMs that use energy and dedicated machinery take up inorganic carbon and concentrate it near Rubisco (Hurd et al. 2009; Brueggeman et al. 2012). I reduce the slope of the relationship between repair and growth from 1 (in the reference scenario) to 0.25 (see Figs 3C and 4C). Cheaper repair leads to a large decrease in the mean damage level of cells in some cases, with about a fourfold reduction in damage at Δ*t* = 0.1 (reference average damage levels over all *α* are 0.078, average damage levels over all *α* for cheap repair are 0.019; variance in damage is <10^−6^ in all cases), and relatively minor changes in damage levels at higher Δ*t*. Along with this, high growth rates of over 1.8 are seen over a wide range of Δ*t* (up to 0.4) and increases in growth relative to the reference scenario occur even at higher Δ*t*. The evolution of this strategy could be measured by applying an exogenous agent that causes oxidative damage to cells and then measuring the actual damage sustained by cells for the same dose of exogenous oxidizing agent in enriched versus reference environments. If repair is cheaper in enriched environments, the cells should show less damage per unit oxidizing agent that they are exposed to.

Finally, the amount of damage incurred per unit increase in growth may be lowered in the new environment. This may be the case if there is a qualitative shift in metabolism, for example, in response to ROS signaling (Munne‐Bosch et al. 2012), or as a direct response to environmental cues (Brueggeman et al. 2012). I explore this by lowering the damage rate from 5 in the ancestral environment to 2 in the new environment (see Figs 3D and 4D). This results in a similar pattern of growth gain as damage tolerance. As expected, reducing the amount of damage per unit growth results in the same pattern and levels of cell damage over the long term as does the reference scenario, as damage still has the same relationship with mortality, but requires higher growth rates to reach a given level of damage. This strategy can be detected by measuring the correlation between growth and damage in both environments.

Regardless of damage tolerance or reduction strategies, growth is higher when the offspring contribution to damage is lower and damage is primarily determined by parents (Δ*t* is smaller). This is because offspring that have a higher growth rate than their parents will not have a much higher damage than offspring with the same or lower growth rates from that same parent, such that higher growth can evolve with less of an increase in damage over time. At the limit where damage is determined almost entirely by the parent, growth can increase by mutation, but damage remains nearly constant over time (in practice, it increases very slowly, as offspring growth rate must make a nonzero contribution to damage in this model). In all cases, more offspring contribution to determining damage results in more offspring mortality events over the course of a simulation, suggesting that in cases where Δ*t* is larger, population extinction becomes increasingly likely.

Generally, rapid growth in an improved environment evolves in this model when damage is primarily determined by the parent. Only cheap repair allows escape from Prodigal Son dynamics over all levels of Δ*t*. None of the strategies result in the evolution rapid growth rates when damage is primarily determined by offspring growth rate (for large Δ*t* of 0.8 and 1.0). However, high growth rates can evolve at intermediate Δ*t* (0.1 and 0.4) when escape strategies are used. The steepness of the damage–mortality relationship affects growth rate evolution more as Δ*t* increases in both the standard and escape scenarios, with a steeper relationship resulting in higher growth rates.

## Discussion

I show that when environments improve, microbial populations may be unable to sustainably increase cell division rates. I show that one reason for this is that damage associated with faster metabolism can force cell division rates to return to ancestral values, or even sink below ancestral values, and show Prodigal Son dynamics. Prodigal Son dynamics can be partially or completely avoided if lineages can evolve damage tolerance, a lower cost of repair, or a lower rate of damage in the new environment. Each of these nonexclusive strategies have different consequences for the properties of the evolved populations. Whether or not strategies to avoid Prodigal Son dynamics emerge will depend on whether an evolutionarily accessible path between the ancestral damage management strategy and an avoidance strategy exists. In addition, the conditions for an avoidance strategy may already be in place if, for example, some quality of the enriched environment allows for higher damage tolerance or cheaper repair even without an evolutionary response. Here, I have investigated Prodigal Son dynamics in terms of growth rate evolution, but propose that other traits correlated with fitness could show the same dynamics under environmental enrichment, where the action of natural selection eventually restores traits to their ancestral values, especially in cases where the response to enrichment is to speed up metabolism (Schaum et al. 2015).

In this model, fitness is determined solely by growth rate and damage, and there are no trade‐offs associated with an increase in damage tolerance and the resulting increase in damage load. This is unlikely to be the case in nondigital organisms, as previous studies have shown that fast‐growing, more damaged cells are poor competitors relative to slower‐growing cells, and less tolerant of environmental change (Schaum and Collins 2014). Thus, increased damage tolerance may be seen in populations where fitness is largely determined by growth rate. This may be the case when populations are growing exponentially without much competition, for example, at the beginning of phytoplankton blooms that are triggered by a nutrient flux, or when a population invades an empty niche. Although damage tolerance without trade‐offs is an unlikely long‐term strategy for speeding up growth, transiently increasing damage tolerance and then transitioning to a slower‐growing or resting stage, where damage can be repaired, may be advantageous. It may also be seen in laboratory studies where single genotypes and relatively permissive environments are used, so that the waiting times for slower‐growing, more competitive mutants to arise may be longer than a single experiment.

On the other hand, it is reasonable to suppose that in an improved environment, resource allocation in cells can be changed, or that there may be qualitative shifts in metabolism that lower the rate of damage. Both of these allow for an increase in growth rate without an increase in damage. While the initial shifts in function may be the result of a plastic response, such as changing CCM activity under high CO_2_ to reallocate resources (Hurd et al. 2009; Raven et al. 2012), there is potential for genetic accommodation over longer timescales, and indeed, results consistent with genetic accommodation have been seen in at least one high CO_2_ experiment (Collins and Bell 2004). I propose that whether or not an evolving population can escape Prodigal Son dynamics and maintain higher growth rates in an enriched environment than it had in the ancestral environment depends on how many different strategies are evolutionarily accessible. For example, if a lineage can evolve damage tolerance, but lacks the genetic or metabolic flexibility to evolve cheaper repair, Prodigal Son dynamics may be inevitable if there are trade‐offs associated with damage tolerance. This is consistent with the re‐evolution of slower growth rates in *Ostreococcus* sp. at high CO_2_ (Schaum and Collins 2014) – this genus has a small genome and although it does have some homologs to the *Chlamydomonas* CCM alongside the machinery for C4 photosynthesis (Peers and Niyogi 2008), it may be too constrained to rapidly evolve cheap repair and divert resources to growth when inorganic carbon is abundant.

This work has potential applications in cases where rapid growth is desired or enriched environments are common, such as growing high‐yield algal cultures for biofuels (Raven and Ralph 2014; Sarkar and Shimizu 2015). In particular, gains in growth or cellular composition realized by manipulating the nutrient ratio of culture media or using very rich media to further speed up already fast‐growing strains may only lead to transient increases in biomass unless the lineages used can escape Prodigal Son dynamics. In this case, studies on genetic manipulation aimed at long‐term yield improvement could be expanded to include upregulating damage repair. Together with empirical work showing that rapidly growing strains evolved in enriched environments often have reduced competitive abilities, it also suggests that high‐biomass strains evolved as single genotypes in enriched environments may be particularly susceptible to contamination by more robust genotypes or taxa. In more natural settings, where environmental enrichment occurs due to fertilizer or waste runoff in aquatic systems, short‐term responses that do not allow sufficient time for Prodigal Son dynamics to occur may overestimate the biotic response to environmental improvement over longer timescales. More generally, a complete understanding of microbial growth rates requires the expansion of both theory and experiments to include responses to environmental improvement alongside more traditional studies focusing on environmental deterioration.

## Data archiving statement

R code for this simulation is available as Supplementary Material.

## Supporting information


**Data S1.** R code for simulation.
