# Supplementary material for: Growth rate evolution in improved environments under Prodigal Son dynamics
Source: Evol Appl. 2016 Sep 28;9(9):1179–88. doi: 10.1111/eva.12403 (PMC5039330; doi:10.1111/eva.12403)
Supplement: Supplementary file 2 [file EVA-9-1179-s002.docx]

R code for simulation.
